# Impact of telehealth on health outcomes and quality of life in the older adults population: a systematic review

**DOI:** 10.3389/fdgth.2025.1708960

**Published:** 2025-12-18

**Authors:** Gonçalo Fernandes, Teodora Figueiredo, Elísio Costa, Luís Coelho, Dirk Loyens

**Affiliations:** 1 ISEP, Polytechnic of Porto, Porto, Portugal; 2 RISE-Health, Competence Center for Active and Healthy Ageing, Faculty of Pharmacy, University of Porto, Porto, Portugal; 3 School of Medicine and Biomedical Sciences, University of Porto, Porto, Portugal; 4 INESC TEC—Institute for Systems and Computer Engineering Technology and Science, Porto, Portugal; 5 ID+, ESMAD, Polytechnic of Porto, Vila do Conde, Portugal

**Keywords:** disease management, health outcomes, health promotion, older adults, quality of life, rehabilitation, systematic review, telehealth

## Abstract

**Background:**

The rapid aging of populations poses major challenges to health and social care systems. Supporting older adults in managing chronic conditions while promoting independence and quality of life requires innovative approaches that extend beyond senior institutional care. Telehealth has emerged as a promising approach to enhance access, continuity, and patient engagement. However, evidence regarding its effectiveness and best practices remains fragmented.

**Objectives:**

This systematic review aimed to synthesize current evidence on telehealth interventions for adults aged 65 years and older, focusing on their effects on health outcomes, quality of life, and well-being.

**Methods:**

A search across three databases in the last five years identified 37 eligible studies, and data analysis was guided by a comprehensive taxonomy. Interventions were diverse, spanning disease management, rehabilitation, health promotion, clinical decision support, and psychological support.

**Results:**

Reported benefits included improved physical function, better chronic disease control, greater health knowledge, and reductions in avoidable hospitalizations. Video-based programs showed greater effectiveness, while telephone-only interventions were most useful when combined with remote monitoring. Adherence was strengthened by professional guidance, caregiver support, and real-time feedback.

**Discussion:**

Despite encouraging findings, evidence remains inconsistent regarding quality-of-life outcomes, cost-effectiveness, and scalability across populations, with many studies limited by small samples, short duration, and methodological heterogeneity. Telehealth holds the potential to complement traditional care for older adults across multiple clinical domains, and future research must adopt consistent and comprehensive reporting practices to strengthen decision-making and ensure that this pathway evolves with patients’ needs.

**Systematic Review Registration:**

PROSPERO CRD420251072656.

## 1 Introduction

The aging population in contemporary mature societies is steadily increasing, representing not only an achievement of humanity, knowledge, and technology through expanded human longevity but also a major public health challenge (1). Although this issue is often viewed from the perspective of individual physiological decline, addressing it should go beyond chronic disease management to also promote quality of life (QOL), independence, and participation in later life, within the framework of active aging (2).

Defined as the process of optimizing opportunities for health, participation, and security, active aging emphasizes the importance of enabling individuals to remain active contributors to society, their community, and their physical and mental well-being across the life course (3). At the same time, older adults are increasingly adopting digital technologies, highlighting the potential of solutions, such as telehealth, to support active aging (4).

Telehealth is a broad concept that extends beyond telemedicine to include not only remote consultations and direct clinical care but also mobile health applications, digital therapeutics, and health education, thus shifting healthcare from episodic and reactive care towards a more continuous, preventive, and holistic model (5). Its delivery can take different formats according to a variety of modalities and purposes, which can be used independently or in combination to improve care effectiveness. The main categories include asynchronous communication, such as transmitting digital images and test results; synchronous communication, involving real-time interactions through audio or video consultations; and remote patient monitoring, which enables patient data collection to track health trends and guide timely interventions (6). These modalities can be implemented through technologies ranging from the most common telephone consultations, particularly valuable for patients with low digital literacy or limited access to the internet or smart devices (7), to videoconferencing, which adds a visual component to virtual visits (8), as well as mobile applications, websites, and SMS messaging (9).

Three main models have emerged to address the diverse context of older adults. Home-based telehealth integrates monitoring technologies and communication tools within the individual's residence, offering flexibility and supporting aging in place strategies, although sometimes limited by usability or literacy barriers (10). Facility-based telehealth is implemented in hospitals, clinics, and nursing homes, where it can expand access to specialists, support staff, and help to reduce avoidable hospitalizations (11, 12). Community-based telehealth takes place in community settings, such as senior centers and congregate housing, where it provides access to health monitoring tools and on-site assistance, often at reduced costs, thereby mitigating disparities in access (10).

The digitalization of healthcare empowers patients to take an active role in managing their own care by connecting with providers anytime and anywhere. Yet, evidence remains limited, with small sample sizes, inconsistent outcome measures, and a lack of consensus on the most effective strategies. While several reviews have examined adherence, satisfaction, usability, and implementation challenges of telehealth interventions in the aging context (13), these aspects may have limited value if not interpreted in relation to their clinical effectiveness, which remains inconsistent and still requires more robust evidence (14).

Given the evolution of technology and the increased maturity of telehealth over the past five years, an updated synthesis is needed to incorporate the latest evidence and capture the advancements in this rapidly evolving field. Recent reviews illustrate both promise and gaps. For instance, video-based rehabilitation and health education have shown benefits, although details about the interventions are often insufficiently reported (15). Telehealth has also been associated with reductions in depressive and anxiety symptoms, yet the available evidence remains limited (16). Similarly, improvements in chronic disease management have been documented, though technology-related challenges and cultural differences in the acceptance of telehealth persist (17, 18). In the field of palliative care, telehealth has emerged as a feasible and acceptable means of supporting shared decision-making among patients, families, and clinicians, but more consistent outcome measures and controlled studies are still needed (19).

Building on the diverse telehealth applications for older adults, this systematic review evaluates their effectiveness to guide future advancements in geriatric care. We intend to capture the range of interventions and their characteristics while connecting them to multidimensional outcomes. This review aims to identify which telehealth interventions have demonstrated the greatest impact on health outcomes, quality of life, and well-being, thereby informing researchers, policymakers, funding agencies, and other stakeholders in the development of future research programs and implementation strategies.

## 2 Methods

The systematic review was conducted in accordance with the Preferred Reporting Items for Systematic Reviews and Meta-Analyses (PRISMA) guidelines (20), ensuring both transparency and methodological thoroughness. This review was registered and accepted on the PROSPERO platform under the registration number CRD420251072656.

### 2.1 Research question

Using the PICO strategy (21), the research question of this review was: How effective are telehealth interventions in improving health outcomes, quality of life, or well-being among older adults?

### 2.2 Objective

This review aims to map the range of telehealth interventions available to older adults and to evaluate their demonstrated impact on well-being, quality of life, and health outcomes regardless of setting.

### 2.3 Search strategy

In May 2025, two reviewers, GF and TF, independently searched and extracted data from PubMed, Scopus, and IEEE Xplore. The final search query was then constructed as: (“telemedicine” OR “telehealth” OR “telerehabilitation” OR “remote consultation” OR “teleradiology” OR “telepathology” OR “distance counseling”) AND (“promot*” OR “prevent*” OR “health promotion” OR “primary prevention” OR “disease prevention” OR “preventive care” OR “preventive health” OR “risk reduction” OR “early detection” OR “early intervention” OR “wellbeing” OR “well-being” OR “quality of life”) AND (“older*” OR “old” OR “geriatr*” OR “aged” OR “ageing” OR “aging” OR “senior*” OR “elder*”) (Supplementary Table 1). Only studies published in the last five years were included, written in English, Portuguese, or Spanish, involving human participants and with full-text availability. The five-year timeframe was chosen to ensure that the review captures the most recent and relevant developments in telehealth technologies and practices, a field that evolved exponentially particularly in response to the transformations triggered by the COVID-19 pandemic. The search was not updated after May 2025.

### 2.4 Study selection

All the obtained entries were reviewed by the same reviewers, who independently assessed the titles, in the first phase, then the abstracts, and finally the full text.

When assessing the titles, articles that met one or more of the exclusion criteria listed below were not considered:

1. Studies that do not focus on Telehealth (i.e., Synchronous Telehealth, Asynchronous Telehealth and Remote Monitoring) or its impact or effectiveness on quality of life, well-being, disease prevention, health promotion, and/or healthcare access, or that do not address the older adult population.
2. Articles written in languages other than English, Portuguese, or Spanish.
3. Study protocols, theoretical papers, conceptual articles, and position papers, as well as non-peer-reviewed publications (e.g., theses and conference abstracts).

During the abstract and full-text analysis, the selection was based on the following inclusion and exclusion criteria:

Inclusion Criteria

1. Study design: Experimental studies, observational studies, qualitative studies, mixed-methods studies, intervention studies, implementation science studies, case studies, and systematic reviews/meta-analyses or scoping reviews.
2. Population: Studies focusing on adults 65 years and older.
3. Intervention: Studies that include data related to the impact of telehealth solutions.
4. Outcomes: Studies that report on healthcare outcomes, quality of life, well-being, disease prevention, and/or health promotion.
5. Publication: Studies published in peer-reviewed journals.

Exclusion Criteria

1. Study protocols, theoretical papers, conceptual papers, and position papers/articles not peer-reviewed (e.g., thesis and conference abstracts).
2. Studies focused on perceptions, evaluation, acceptance, feasibility, expectations, usability, use, development, barriers, preferences, adoption, characteristics, and implementation of telehealth solutions.
3. Studies that do not focus on telehealth solutions (i.e., Synchronous Telehealth, Asynchronous Telehealth and Remote Monitoring).
4. Studies that do not focus exclusively on older adults (i.e., studies that include mixed populations without a focus on older adults).

### 2.5 Data extraction

Data extraction was performed by two independent reviewers (GF and TF) using two standardized forms, one for review articles and another for original studies. Regarding review articles, data extraction included: authorship, year of publication, country, number of studies included, review objective, intervention details (types of interventions included and comparators), outcomes assessed, and the main findings across studies.

For original articles the following information was captured: authorship, year of publication, country, study design, study aim, population characteristics (including health conditions, age, and sample size), intervention details (duration, frequency, and delivery modalities), outcomes measured, and their significant changes as the main findings.

**Figure 1:**
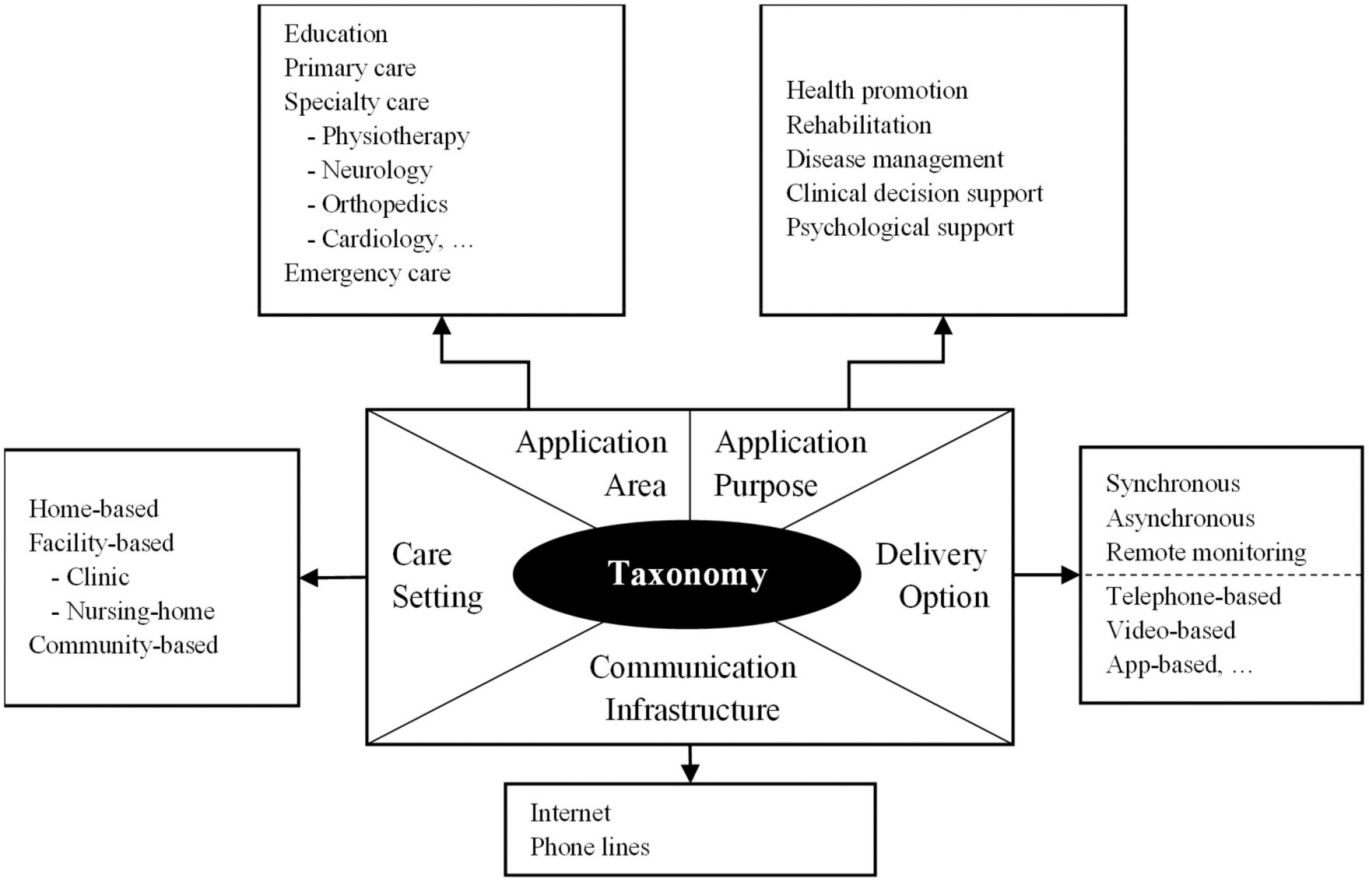
Data extraction template (adaptation of the Tulu et al. (22) taxonomy).

Content analysis was guided by an adaptation of the taxonomy developed by Tulu et al. (22), which provides a structured guide for identifying telehealth interventions according to five dimensions (Figure 1). The first dimension, application area, refers to the medical domain of the intervention. The second, application purpose, identifies the main goal of the intervention. The third, care settings, refers to the physical location of the patient. The fourth dimension, delivery options, covers both the delivery modalities and the technologies used. Finally, the fifth dimension, communication infrastructure, concerns the channels used for the transmission and reception of data or information.

A meta-analysis was not conducted due to the substantial heterogeneity across interventions, populations, study designs, and outcome measures.

### 2.6 Quality assessment

The methodological quality of the included studies was assessed using the Joanna Briggs Institute (JBI) Critical Appraisal Tools, with specific validated checklists applied according to each study design. These tools were selected as applicable across a wide range of study types within systematic reviews, enabling consistent quality appraisal, and are widely used in health research (23).

Each study was evaluated with the corresponding JBI checklist, where items were scored as 1 (“Yes”) or 0 (“No,” “Unclear”, or “Not applicable”). An overall quality score was calculated as the proportion of “Yes” responses relative to the total number of items. Based on these scores, studies were classified as high quality (≥75%), moderate quality (50%–74%), or low quality (<50%) (see Supplementary Table 2).

Articles were not excluded based on the JBI score, as the objective was only to examine the rigor of the included studies. However, it is important to note that these checklists remain subjective tools and that quality appraisal was conducted by two independent reviewers (GF and TF), which may have introduced bias and should be considered when interpreting the results. Overall, 10 studies were rated as high quality, 13 scored moderate quality, and 14 as low quality, with the general methodological standard considered moderate.

## 3 Results

### 3.1 Search results

**Figure 2:**
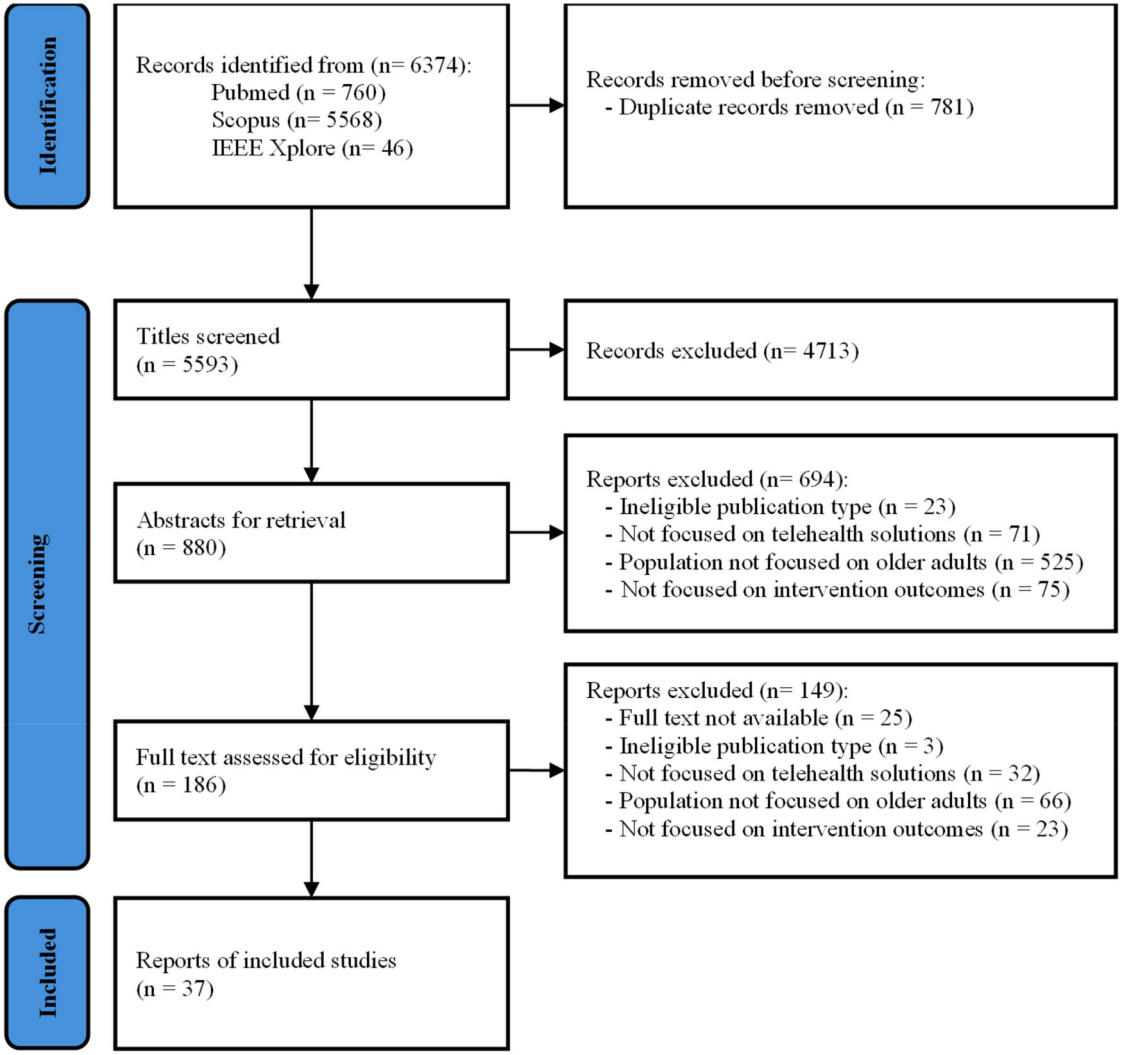
PRISMA flowchart.

From the initial 6,374 identified articles, duplicates were removed, leaving 5,593 articles for the first stage of the screening process based on titles. Of these, 4,713 articles were excluded at this stage, leaving 880 papers for abstract screening. After further analysis, 186 articles underwent full-text analysis, resulting in 37 articles (6 reviews and 31 original articles) being chosen to be included in the review. The entire selection process, along with reasons for exclusion at various stages, is visually summarized in a PRISMA flowchart (Figure 2).

The agreement between the two independent reviewers was assessed using Cohen's Kappa coefficient. Decisions to include or exclude articles were coded as binary (included = 1, excluded = −1). The Kappa value was calculated for the three screening phases: in the title phase, the resulting value was 0.4559, indicating moderate agreement according to Landis and Koch's classification (24). In the abstract screening phase, the Kappa value was 0.4111, indicating moderate agreement between reviewers, and the full-text phase was 0.9046, indicating substantial agreement.

### 3.2 Characteristics of the included articles

#### 3.2.1 Findings from the reviews

The six included reviews covered studies conducted worldwide, totaling 59 original articles. Notably, all reviews included studies published only up to 2022 (Supplementary Table 3). Regarding study design, four were systematic reviews (25–28), two of which included meta-analyses (25, 26); one was a literature review (29), and one was a scoping review (30).

Five of the six reviews concluded that telehealth interventions offer various therapeutic effects for older adults, particularly in relation to physical health, QOL, healthcare service utilization, and overall health management.

Two reviews (25, 26), assessed exercise programs delivered via telehealth, reporting general improvements in physical function, physical activity, mobility, strength, balance, frailty status, and fall incidence. While Dawson et al. (25) found mixed results regarding QOL, Esfandiari et al. (26) reported no significant effects. The latter described mostly telephone-based interventions targeting fall prevention and physical activity through self-management strategies. In contrast, Dawson et al. (25) included primarily physiotherapist-led interventions, delivered through synchronous video sessions or asynchronous formats.

Dawson et al. (25) also reported high adherence rates, with a median of 86%, attributing this to the combination of professional support with monitoring and feedback mechanisms, which enabled real-time performance evaluation and clear exercise instructions. Adherence was further supported by initial participant training, ongoing technological assistance, the convenience of home-based delivery, and the flexibility of asynchronous programs that allowed participants to exercise on their own schedule, with mixed technological hesitancy impact. Thus, recommendations were made to explore how high adherence might be leveraged to increase intervention intensity and improve outcomes.

Esfandiari et al. (26) noted that although telehealth may yield long-term savings for healthcare systems, implementation may be challenged by high upfront costs, highlighting the need for further research into the cost-effectiveness of such interventions.

A review focusing on telecare, mainly telemonitoring and telerehabilitation interventions, reported improvements in QOL across physical, mental, and social domains (27). Telemonitoring typically involved vital sign tracking, automated alerts, and teleconsultations, supporting the management of chronic conditions such as diabetes, chronic heart failure (CHF), and chronic obstructive pulmonary disease (COPD). Telerehabilitation was presented as a viable alternative to in-person rehabilitation, particularly in stroke and COPD patients, with videoconferencing contributing to significant physical improvements and QOL gains. However, usability issues were reported, underscoring the need for user-friendly, tailored solutions that reflect older adults' abilities, skills, and preferences.

Another review examined interventions combining health coaching with remote monitoring, reporting positive outcomes, particularly improving physical activity levels and better control of hemoglobin and body weight in diabetes patients (29). Most studies involved telephone-based support, initial training, and scheduled or reactive follow-ups. Given the mixed results with both telephone calls and electronic media, the authors suggested that effectiveness may not depend on the communication medium and encouraged the exploration of digital platforms. Most interventions employed human coaches, prompting interest in the potential of automated coaching to enhance cost-efficiency and allow 24/7 support. Additionally, patient willingness to engage in coaching emerged as a key factor, with calls for research involving less receptive populations.

One review assessed telephone-based follow-up after emergency department (ED) discharge and found no significant impact on return visits, hospitalizations, mortality, or medication adherence (28). Although patients were generally accessible by phone, many declined participation, suggesting that this type of follow-up may be perceived as unnecessary. Nonetheless, the authors emphasized older adults' need for social and emotional support post-discharge, recommending further investigation into the potential benefits of such interventions.

Finally, one review focused on telehealth-delivered palliative care in nursing homes for recently hospitalized patients, reporting benefits such as increased goals-of-care discussions and reduced acute care use, alongside mixed findings for QOL (30). Most interventions involved video consultations between palliative care teams and residents and/or their surrogate decision-makers, addressing advance care planning and both physical and psychological needs. Many studies adopted a team-based care approach, either involving nursing home staff and physicians or broader multidisciplinary teams, supporting timely access to specialized care and more effective care planning. These strategies were associated with reduced healthcare utilization, although some barriers such as technological limitations and high upfront costs were noted.

Regarding care settings, most of the reviewed interventions were delivered in home-based contexts, such as remote exercise programs, telemonitoring of vital signs and activities, health coaching, and follow-ups (25–29). Some interventions were implemented in facility-based settings, particularly nursing homes, mainly in the context of palliative care or rehabilitation support (25, 29). Only one community-based study was identified (27), reporting a physiotherapist-led telerehabilitation intervention via video conferencing, conducted in a community center, which showed significant improvements in physical functioning QOL, social functioning, and mental health problems.

Furthermore, one study included in the review by Dawson et al. (25) recruited participants through a community center, where the intervention was established via videoconferencing, in conjunction with home-based exercises. This approach reported the highest levels of reach and adherence among the included studies, suggesting that engaging older adults through familiar and socially trusted environments may enhance their participation and commitment, even when the intervention is delivered remotely.

#### 3.2.2 Findings from the original articles

The included studies were published between 2020 and 2025, with half [*n* = 15; (31–45)] appearing after 2022, providing more recent evidence to complement the findings from previous reviews. Most studies were conducted in Europe (33, 36, 40, 41, 46–55), followed by Asia [*n* = 11; (34, 35, 38, 43–45, 56–60)]. The main characteristics and conclusions are summarized in Supplementary Table 4.

Regarding study design, almost half were randomized controlled trials (RCTs) [*n* = 13; (35, 38, 40–43, 45, 48, 49, 55, 58, 60, 61)]. Quasi-experimental studies represented 29.0% [*n* = 9; (33, 36, 39, 44, 46, 50, 51, 54, 57)], while prospective cohort studies, case studies, and other designs accounted for the remaining 29.0% [*n* = 9; (31, 32, 34, 37, 47, 52, 53, 56, 59)].

Across all studies, a total of 4,144 participants were included. Only two studies (6.5%) reported mean or median participant ages between 65 and 69 years (52, 56), while more than half (54.8%) included participants between the ages of 70 and 79 [*n* = 17; (31, 32, 37, 41, 43–46, 48, 50, 51, 54, 55, 57–60)]. Seven studies (22.6%) included participants ≥80 years (33, 34, 36, 42, 49, 53, 61), and the remaining five studies (16.1%) either specified a threshold ≥65 years or included younger participants while focusing on older adults (35, 38–40, 47).

Many interventions aspired to improve healthcare accessibility, with ten studies (32.3%) conducted during the COVID-19 pandemic to ensure continuity of care during confinement (33, 41, 42, 46, 47, 55–58, 60). Most targeted disease management with specialty care, often through videoconference-based home interventions (Table 1).

**Table 1:** Synthesis of the content analysis of the reviewed original articles organized in accordance with the used taxonomy.

Dimension	Characteristic	*n* (%)
Application purpose	Disease management (32, 34, 36, 39, 44, 45, 47, 48, 51–54, 57, 61)	14 (45.1)
	Rehabilitation (35, 38, 40, 41, 44, 46, 50, 55, 56, 58–60)	12 (38.7)
	Health promotion (31, 37, 39, 43, 45, 57, 61)	7 (22.6)
	Clinical decision support (32, 36, 42, 47, 49, 53)	6 (19.4)
	Psychological support (33, 37, 48)	3 (9.7)
Application area	Education (31, 37, 39, 43, 45, 57, 61)	7 (22.6)
	Specialty care	23 (74.2)
	- Physiotherapy (35, 38, 40, 41, 44, 46, 50, 55, 56, 58–60)	12 (38.7)
	- Neurology (33, 35, 37, 41, 52, 56–58, 61)	9 (29.0)
	- Cardiology (53, 54, 59)	3 (9.7)
	- Orthopedics (34, 40, 44)	3 (9.7)
	- Endocrinology (36, 47)	2 (6.4)
	- Oncology (32, 50)	2 (6.4)
	- Pulmonology (54)	1 (3.2)
	Primary care (32, 48, 49, 51)	4 (12.9)
	Emergency care (42)	1 (3.2)
Delivery options	Synchronous	26 (83.8)
	- Videoconference (31–33, 37–40, 42–46, 48–50, 55–58, 60, 61)	21 (67.7)
	- Telephone (34, 36, 38, 42, 44, 47, 52, 57, 59–61)	11 (35.5)
	Asynchronous	4 (12.9)
	- Email (47)	1 (3.2)
	- App (35)	1 (3.2)
	- Video tutorials (35, 41)	2 (6.5)
	- SMS (54)	1 (3.2)
	Remote monitoring	13 (41.9)
	- Wearable (36, 41, 43, 45, 46, 50)	6 (19.4)
	- Peripheral devices (36, 41, 44, 48, 49, 51, 53, 54, 56, 59)	10 (32.3)
Communication infrastructure	Internet (31–33, 35–51, 53–61)	29 (93.5)
	Phone lines (34, 36, 38, 42, 44, 47, 51, 52, 54, 57, 59, 60)	12 (38.7)
Care settings	Home-based (31, 32, 34, 35, 37–41, 43–48, 50–61)	27 (87.1)
	Facility-based	4 (12.9)
	- Nursing-home (36, 42, 49)	3 (9.7)
	- Hospital/Clinic (33)	1 (3.2)
	Community-based	0

##### 3.2.2.1 Application purpose

Telehealth was primarily used for disease management support in nearly half of the studies, usually through follow-ups or remote monitoring, aiming to increase adherence to care plans. In some cases, this was combined with clinical decision support [*n* = 4; (32, 36, 47, 53)] or health promotion strategies [*n* = 4; (39, 45, 57, 61)].

The second most frequent purpose was rehabilitation, generally as a standalone intervention, focusing on physical functioning and physical activity in daily living or in pre- and post-operative contexts. Health promotion interventions were also reported both independently (as primary/secondary prevention) and combined with disease management (tertiary prevention), often aligned with concepts of active aging and aging in place.

Other interventions focused on supporting clinical decision-making in providing timely care or preventing avoidable hospitalizations particularly in nursing home residents. Psychological support appeared across multiple studies, but was the primary aim in three, mostly as a way of reducing social isolation (Table 1).

##### 3.2.2.2 Application area

Physiotherapy interventions were the most frequent, targeting improvements in physical activity, muscle strength, balance, gait, physical functioning, and post-operative recovery, often among frail, homebound, or confined older adults. These were sometimes linked to neurological, orthopedic, or cardiac conditions.

Neurological interventions targeted Parkinson's disease, Alzheimer's disease, and dementia. Parkinson's interventions focused on gait, balance, and strength; Alzheimer's programs addressed cognition, depression, and independence; and dementia interventions frequently combined education with psychological support, aiming to reduce loneliness, improve QOL, and optimize care plans.

Education interventions mainly promoted health literacy, guiding older adults to manage their health and adopt beneficial daily habits. Primary care interventions were typically team-based, supporting clinical decision-making through monitoring and follow-ups, enabling timely referrals to specialty care, and contributing to reduced hospitalizations. Other areas included orthopedics (pre/post-operative exercises, osteoporosis treatment), cardiology/pulmonology (CHF, hypertension, and COPD management with remote monitoring), oncology (supportive care before chemotherapy or surgery), endocrinology (follow-up and treatment plan updates) and emergency care in nursing homes (virtual support for acute episodes, preventing unnecessary hospitalizations) (Table 1).

##### 3.2.2.3 Delivery options

Most interventions employed synchronous telehealth, particularly real-time videoconferencing platforms (Zoom, WhatsApp, Google Meet, Skype). Telephone calls were also widely used (over one third of studies), either as the sole mode, in combination with videoconferencing, or as comparators.

Nearly half of the studies used remote monitoring, typically with peripheral devices (e.g., stethoscopes, oximeters, weight scales, sphygmomanometers, digital pens, insulin pens). Wearables were also common, tracking physical activity, vital signs, glucose, and sleep. These often relied on connected platforms such as TV-based or box hubs for Bluetooth devices, a virtual reality/treadmill system, or apps linked to peripherals and wearables. Remote monitoring was usually complemented by synchronous communication.

Asynchronous telehealth was the least frequent. Examples included tutorial exercise videos, mobile apps with educational content and adverse event reporting, as well as email or SMS messaging (Table 1).

##### 3.2.2.4 Communication infrastructure

Almost all interventions required internet connectivity, either directly (videoconferencing) or indirectly (remote monitoring). Almost one third also relied on telephone communication, often in hybrid formats. Only two studies relied exclusively on telephone as the primary channel (Table 1).

##### 3.2.2.5 Care settings

Most interventions were delivered in home settings, with only four studies describing facility-based telehealth in nursing-homes or hospitals. No intervention was reported as being delivered in community-based settings, although some were rooted in community care frameworks (Table 1).

##### 3.2.2.6 Additional characteristics

Recruitment most often occurred in healthcare environments, such as hospitals, clinics, or following hospital discharge [*n* = 18; (32–35, 40, 41, 44, 47, 48, 50–54, 56, 58, 59, 61)]. Others recruited participants through community channels, including advertisements, surveys, journals, senior centers, or nursing homes [*n* = 13; (31, 36–39, 42, 43, 45, 46, 49, 55, 57, 60)]. Some studies described more restrictive inclusion approaches, recruiting only participants who already had internet access and basic technological skills (e.g., use of mobile phones, videoconferencing, or digital platforms) [*n* = 9; (31, 35, 37, 40, 43, 45, 51, 55, 58)].

The total duration of interventions ranged from 25 days to 14 months, with a median of 10 weeks. Among the studies that reported these details, the median intervention frequency was three sessions per week, with a median session duration of approximately 40 min.

Regarding professionals involved, almost half the interventions were led by physiotherapists [*n* = 10; (35, 38, 40, 44, 46, 50, 55, 56, 58, 60)], occupational therapists [*n* = 4; (31, 39, 50, 61)], or therapists without further specification [*n* = 2; (37, 41)]. Six interventions (19.4%) were delivered by multi-specialist teams (32, 33, 44, 47, 48, 59), while four (12.9%) were led by nurses (34, 42, 45, 52). Others were directed by general practitioners [*n* = 2; (51, 53)] or endocrinologists [*n* = 1; (36)].

Support structures were also reported in several studies. Half the interventions included some degree of caregiver involvement, particularly in dyadic approaches [*n* = 15; (32, 33, 35, 38, 41, 44, 47, 48, 50–53, 57, 58, 61)]. Dyadic telehealth interventions, explicitly designed to benefit both patients and caregivers, were described in five studies (33, 48, 50, 57, 61). Their aims included strengthening patient-caregiver relationships, reducing caregiver burden, improving self-efficacy, and enhancing the mental health, QOL, and well-being of caregivers.

Five studies (16.1%) described initial in-person instructions or training to facilitate engagement with the intervention (40, 41, 46, 59, 61), and four (12.9%) reported support by staff within hospital or nursing homes (36, 38, 42, 49).

##### 3.2.2.7 Outcomes

The 31 studies used a wide range of variables to measure the outcomes of their interventions (Supplementary Table 5). Physical functioning and rehabilitation outcomes were assessed in eleven studies (35.5%), focusing on physical activity, performance, balance, gait, and falls (35, 38, 40, 43, 46, 50, 55, 56, 58–60). Among the most frequently used measures were the Timed Up and Go Test, the Five Times Sit-to-Stand Test, and the Berg Balance Scale.

Clinical and disease management outcomes were evaluated in fifteen studies (48.4%). These included control of vital signs such as blood pressure and glycaemia, and sleep parameters, disease-specific measures for Parkinson's disease, Alzheimer's disease, coronary conditions and post-surgical recovery, anti-osteoporosis treatment, nutritional adherence, and changes in ED utilization (34–36, 39, 41, 42, 44–49, 52, 53, 59).

Cognitive and psychosocial outcomes were reported in thirteen studies (41.9%). Cognitive functioning was assessed with a variety of standardized instruments, alongside measures of depression, anxiety, stress, resilience, loneliness, and aging perceptions (31–33, 37, 41, 48, 49, 51, 55, 57, 58, 60, 61). Notably, the Geriatric Depression Scale was applied in seven studies (32, 33, 48, 49, 51, 58, 60).

Finally, QOL and well-being outcomes were measured in fifteen studies (48.4%). Health-related quality of life (HRQOL) was the most frequently assessed domain, typically using standardized questionnaires, with EQ-5D being the most common. Several studies also evaluated independence in daily living activities, reflecting the functional impact of interventions (32, 33, 35, 39, 40, 45, 46, 48, 49, 51, 54, 55, 57, 58, 61).

The significant outcomes reported were diverse across telehealth application areas.

General educational interventions increased health knowledge and skills (31), helped patients set and prioritize health goals, thereby improving chronic disease management (39), promoted higher levels of physical activity and reduced sedentariness (43), and improved vital signs control as well as QOL (45).

Neurological conditions were the most common, with interventions yielding physical, cognitive, psychosocial and QOL benefits. In Parkinson's disease patients, physical therapy improved gait (35, 56), balance (35, 56) muscle strength (35), and QOL (35), while reducing disability (35). A follow-up program also reduced non-motor symptoms such as anxiety and sleepiness, contributing to fewer falls (52). For patients with Alzheimer's disease and other dementias, cognitive exercises improved overall cognition (33, 58), particularly language (41), as well as memory (58), functional independence (58), QOL (33), and dementia status (33). Psychosocial benefits included reductions in anxiety (33, 58) and depression (58). Physical exercises further supported physical performance (58). Educational programs also enhanced cognition and QOL (57), reduced stress and loneliness, improved resilience, and aging perceptions (37), and promoted better patient-caregiver relationships (61).

General physiotherapy interventions improved physical activity, sleep parameters and QOL (46), physical performance (38, 55, 60), balance (38, 55, 60), gait (38, 60), muscle strength (38, 60), reduced falls (38, 55), and decreased anxiety (55).

Orthopedic conditions included physical exercise that increased physical performance and QOL among pre-surgery patients (40), while also reducing complications, anxiety, and increasing functional independence after surgery (44). An adherence-related follow-up intervention improved compliance with anti-osteoporosis treatments and supplementation (34).

Cardiac and pulmonary interventions showed that remote monitoring of vital signs and health status led to improved blood pressure control among hypertensive patients (53), and HRQOL in CHF and COPD patients (54). Exercise interventions increased physical performance, ejection fraction, and reduced readmissions in CHF patients (59).

Oncological interventions demonstrated benefits before chemotherapy, with a patient-centered program improving HRQOL, independence, and depression symptoms (32). Pre-operative physical exercise also enhanced post-surgical performance (50).

Endocrinological interventions were effective in increasing adherence to follow-up procedures (47) and reducing hypoglycemic events while improving glycemic control in diabetes patients (36).

Primary care interventions, delivered by multi-specialist teams, led to timely referrals to other specialties (32), improved independence and nutritional status (48), reduced unplanned hospitalizations of nursing-home residents (49), and enhanced the mental component of HRQOL (51).

Finally, in emergency care, a telehealth follow-up program reduced ED presentations by supporting staff in managing acute events in nursing-home residents (42).

## 4 Discussion

### 4.1 Main findings

The exponential expansion of telehealth in recent years has offered new ways of delivering care to older adults, with the potential to support active aging in parallel with the growing technology adoption within this population (4). However, previous reviews have examined aspects such as adherence, satisfaction, usability, and implementation challenges, often without linking these aspects to clinical effectiveness (13, 14), resulting in fragmented and inconsistent evidence that is insufficient to inform decision-making. The present systematic review, covering 6 reviews and 31 original articles, highlights the diversity of telehealth interventions for older adults and their potential to complement traditional care across multiple clinical modalities.

Reported benefits in the reviews spanned physical health, QOL, and chronic disease management, although results were variable. Exercise interventions via videoconferencing tended to produce clearer gains in physical function (25, 27), than telephone-based self-management (26), emphasizing the role of professional involvement and delivery format. While video-based telerehabilitation has been identified by some as a potential alternative to in-person rehabilitation presenting favorable evidence (27), defending it as a full replacement remains an overstatement given the mixed effectiveness findings and limited evidence (25). Telemonitoring showed more consistent improvements in disease management but raised usability concerns, pointing to the importance of tailoring interventions to older adults' capacities. Other modalities also revealed mixed findings: telephone-based health coaching combined with remote monitoring supported physical activity and chronic disease management, although effectiveness relied heavily on motivation (29); by contrast, telephone follow-up after ED discharge appeared poorly aligned with patients' needs (28), whereas telehealth-delivered palliative care in nursing homes facilitated goals-of-care discussions, reduced acute care use, and improved multidisciplinary collaboration (30).

The need for professional involvement and concerns over its adaptability across conditions and populations, suggest that telehealth may be more effective when positioned as an extension of specialized care rather than a fully autonomous solution. These findings are complemented by the original studies, nearly half published after 2022, reflecting the rapid expansion of telehealth during and after the COVID-19 pandemic. Interventions were heterogeneous in purpose and scope, covering disease management, rehabilitation, health promotion, clinical decision support, and psychosocial support.

Disease management interventions frequently combined telephone follow-ups with remote monitoring, producing benefits such as improved cognitive functioning and psychological outcomes in neurological patients, better blood pressure and glycemic control, increased adherence to endocrine and osteoporosis treatments, and improved preparedness for oncology patients. Videoconferencing showed advantages over telephone delivery in provider-patient connection (47) and outcomes (33, 48), suggesting the need to clarify when and for whom video communication should be prioritized over telephone.

Rehabilitation, mainly physiotherapy-led via videoconferencing, improved physical performance, gait, balance, fall prevention, and functional independence, particularly among frail or homebound older adults and patients with Parkinson's disease. Pre- and post-operative programs enhanced recovery after hip and cancer surgeries. While some studies reported equivalence with in-person programs (38, 55), asynchronous video tutorials were less effective (35) and telephone follow-ups inferior to video-based rehabilitation (44), reinforcing the importance of real-time supervision.

Health promotion interventions, mostly video-based, addressed active aging, lifestyle, and community living, improving knowledge, aging perceptions, sedentariness, and patient-caregiver relationships. Combined video and telephone interventions appeared superior to telephone alone (57), while video-based education proved comparable to home visits (61), indicating its feasibility for preventive care. Some studies did not clearly assess whether knowledge gains translated into sustained behavioral changes (31), but when paired with remote monitoring and disease management, health promotion interventions may be most effective as both a source of health knowledge and a motivational complement to broader care strategies.

Clinical decision support, primarily in nursing homes, helped staff manage chronic conditions and acute cases, reduced unplanned hospitalizations, and enabled referrals, strengthening care coordination. Psychological support, showed promise in reducing loneliness, stress, and depression among older adults with dementia or chronic conditions, highlighting a priority area given the prevalence of social isolation.

Despite positive findings, QOL and well-being outcomes were inconsistent, possibly due to challenges in data collection (49), short interventions and low engagement (35), heterogenous methodologies and small samples (39), or measurement tools limitations (58), indicating that the evidence in this domain should be interpreted with caution. Methodological and clinical heterogeneity of studies, including the variability of intervention purposes, delivery formats, inclusion criteria, session frequency, level of supervision, and outcome measures, may further explain divergent results and hinder comparability. Additional limitations, such as the lack of control groups, small sample sizes, and short duration of some interventions, restrict the validity of causal interpretations and the generalizability of findings.

Most interventions were home-based, often recruiting patients from hospitals and involving caregiver support or technical assistance. Facility-based interventions, mainly in nursing homes, supported staff in clinical decision-making and resident care despite challenges such as staff shortages (49) and difficulty engaging residents with cognitive impairment (36). No purely community-based programs were found, though some resembled this approach. While most interventions were individual, some were delivered to groups, with authors noting the feasibility of supervising multiple participants simultaneously (56). Two physiotherapist-led exercise programs streamed from welfare centers were effective (38, 60), although participants attending in person benefitted from social interaction and on-site support, factors that may enhance motivation and adherence (38). These observations suggest that the effectiveness of remote interventions is context-dependent, impacted by available support and social interaction, which could limit their generalizability to fully independent settings.

Adherence emerged as an effectiveness determinant in both reviews and original studies, with professional and caregiver support, real-time feedback, flexible scheduling, technological assistance, and initial training identified as facilitators. Additional strategies included motivation calls (60) and self-motivation techniques (41). Highlighted gaps were also similar, pointing to inconsistent QOL outcomes, scarce cost-effectiveness evidence, and inclusion of primarily digitally literate or caregiver-supported older adults.

### 4.2 Strengths and limitations

This review provides a comprehensive picture of the promise of telehealth for older adults aged 65 or older, being the first to the best of knowledge to map diverse intervention scenarios, delivery models, and their most common outcomes. The inclusion of three databases enabled a substantial and balanced synthesis. Nonetheless, some limitations must be acknowledged. The exclusion of studies in languages other than English, Portuguese, and Spanish, as well as the restricted time frame, may have introduced language bias and led to the omission of relevant studies. Limiting the search to peer-reviewed literature may have also excluded valuable grey literature, which is particularly relevant in such a rapidly evolving field. Excluding studies with mixed populations to narrow the analysis may have further omitted evidence of effective interventions. In addition, focusing on statistically significant outcomes was intended to ensure clinical relevance and methodological rigor, but this approach may have overrepresented positive findings. By excluding non-significant findings, which may still carry exploratory value, the synthesis risks underrepresenting the full spectrum of evidence and unintentionally amplifying the perceived strength of some associations. Furthermore, the moderate methodological quality and the significant heterogeneity of included studies complicate comparisons and affect interpretation of results, limiting generalizability. At the same time, this diversity reflects the range of telehealth applications, providing insights that may be applicable across different clinical contexts.

### 4.3 Recommendations

While the evidence supports the role of telehealth across multiple clinical domains, its effectiveness depends on careful design, professional involvement, and equitable access. The findings of this review suggest that telehealth is most beneficial when used as an extension of existing care, particularly when interventions are synchronous, supervised, and supported by caregiver or technical assistance, especially in rehabilitation and remote monitoring contexts. However, benefits may be limited for older adults with insufficient digital literacy, highlighting a risk of digital exclusion and inequities in telehealth access. More inclusive approaches are essential to ensure that these groups are not left behind. These may include combining telehealth with in-person care within hybrid models, providing initial in-person training, offering group-delivered sessions, and using low-tech alternatives and intuitive interfaces.

Future research should prioritize controlled trials, cost-effectiveness analyses, and the investigation of optimal conditions for individual vs. group delivery. In addition, studies exploring older adults' perceptions, preferences, and experiences with telehealth are essential to complement quantitative outcomes, ensuring that the telehealth path evolves with patients, not the other way around. Given the integration of artificial intelligence and broader digitalization in healthcare, implementation should be guided by robust evidence to ensure interventions are effective. Moreover, consistent and comprehensive reporting, using standardized clinical research strategies and outcome measures, is needed to better support decision-making.

## 5 Conclusion

This systematic review highlights how telehealth can complement traditional care for older adults across multiple domains, including disease management, rehabilitation, health promotion, clinical decision-making, and psychological support. It allowed us to map the diversity of modalities, their interconnections, and the contextual factors that influence their effectiveness. Delivery formats and professional involvement were key determinants of effectiveness, underlining the importance of tailoring the interventions to the characteristics of each modality. Findings suggest that supportive structures, group delivery, and peer interaction may enhance engagement and outcomes. Future research should integrate clinical trials with studies on user perceptions, usability, and cost-effectiveness, while also adopting consistent reporting standards to enable comparability and informed decision-making. Considering the potential benefits of hybrid delivery models and inclusive approaches for seniors, especially those with limited digital literacy or health-related restrictions, can further enhance the accessibility and impact of telehealth. Only through such inclusive and comprehensive approaches can telehealth evolve into a truly effective response to the needs of older adults, contributing to more sustainable and connected models of aging.

## Data Availability

The original contributions presented in the study are included in the article/Supplementary Material, further inquiries can be directed to the corresponding author.
