# Supplementary material for: Impact of telehealth on health outcomes and quality of life in the older adults population: a systematic review
Source: Front Digit Health. 2025 Dec 18;7:1708960. doi: 10.3389/fdgth.2025.1708960 (PMC12756505; doi:10.3389/fdgth.2025.1708960)
Supplement: Supplementary file 1 [file Datasheet1.pdf]

Supplementary Table 1. Overview of the search strings and the number of articles retrieved per database. The search was performed with a five-year timeframe. The search was performed in May 2025.

Database	Search strings	Results (number of articles retrieved, n)
**PubMed**	(((telemedicine[Title/Abstract] OR telehealth[Title/Abstract] OR telerehabilitation[Title/Abstract] OR "remote consultation"[Title/Abstract] OR teleradiology[Title/Abstract] OR telepathology[Title/Abstract] OR "distance counseling"[Title/Abstract])) AND ((promot*[Title/Abstract] OR prevent*[Title/Abstract] OR "health promotion"[Title/Abstract] OR "primary prevention"[Title/Abstract] OR "disease prevention"[Title/Abstract] OR "preventive care"[Title/Abstract] OR "preventive health"[Title/Abstract] OR "risk reduction"[Title/Abstract] OR "early detection"[Title/Abstract] OR "early intervention"OR wellbeing[Title/Abstract] OR "well-being"[Title/Abstract] OR "quality of life"[Title/Abstract]))) AND ((older*[Title/Abstract] OR old[Title/Abstract] OR geriatr*[Title/Abstract] OR aged[Title/Abstract] OR ageing[Title/Abstract] OR aging[Title/Abstract] OR senior*[Title/Abstract] OR elder*[Title/Abstract]))	n = 760
**Scopus**	TITLE-ABS-KEY ((telemedicine OR telehealth OR telerehabilitation OR "remote consultation" OR teleradiology OR telepathology OR "distance counseling") AND (promot* OR prevent* OR "health promotion" OR "primary prevention" OR "disease prevention" OR "preventive care" OR "preventive health" OR "risk reduction" OR "early detection" OR "early intervention" OR wellbeing OR "well-being" OR "quality of life") AND (older* OR old OR geriatr* OR aged OR ageing OR aging OR senior* OR elder*))	n = 5568
**IEEE Xplore**	("Publication Title":telemedicine OR "Publication Title":telehealth OR "Publication Title":telerehabilitation OR "Publication Title":"remote consultation" OR "Publication Title":teleradiology OR "Publication Title":telepathology OR "Publication Title":"distance counseling") AND ("Publication Title":promot* OR "Publication Title":prevent* OR "Publication Title":"health promotion" OR "Publication Title":"primary prevention" OR "Publication Title":"disease prevention" OR "Publication Title":"preventive care" OR "Publication Title":"preventive health" OR "Publication Title":"risk reduction" OR "Publication Title":"early detection" OR "Publication Title":"early intervention"OR wellbeing OR "Publication Title":"well-being" OR "Publication Title":"quality of life") AND ("Publication Title":older* OR "Publication Title":old OR "Publication Title":geriatr* OR "Publication Title":aged OR "Publication	n = 0 + 46

	Title":ageing OR "Publication Title":aging OR "Publication Title":senior* OR "Publication Title":elder*)<br><br>+<br><br>("Abstract":telemedicine OR "Abstract":telehealth OR "Abstract":telerehabilitation OR "Abstract":"remote consultation" OR "Abstract":teleradiology OR "Abstract":telepathology OR "Abstract":"distance counseling") AND ("Abstract":promot* OR "Abstract":prevent* OR "Abstract":"health promotion" OR "Abstract":"primary prevention" OR "Abstract":"disease prevention" OR "Abstract":"preventive care" OR "Abstract":"preventive health" OR "Abstract":"risk reduction" OR "Abstract":"early detection" OR "Abstract":"early intervention"OR wellbeing OR "Abstract":"well-being" OR "Abstract":"quality of life") AND ("Abstract":older* OR "Abstract":old OR "Abstract":geriatr* OR "Abstract":aged OR "Abstract":ageing OR "Abstract":aging OR "Abstract":senior* OR "Abstract":elder*)	
Total		n = 6374
Total after removing duplicates		n = 5593

Supplementary Table 2. Quality assessment of the included articles using the JBI critical appraisal checklists.

**Publication**	**Study Design**	**Critical Appraisal Tool Used**	**Score**	**Quality (high/moderate/low)**
(Dawson et al., 2024)	Systematic review and meta-analysis	Checklist for Systematic Reviews and Research Syntheses	10/11	High
(Esfandiari, Miller & Ashe, 2021)	Systematic review and meta-analysis	Checklist for Systematic Reviews and Research Syntheses	10/11	High

(Eslami Jahromi & Ayatollahi, 2023)	Systematic review	Checklist for Systematic Reviews and Research Syntheses	10/11	High
(Markert et al., 2021)	Literature review	Checklist for Systematic Reviews and Research Syntheses	3/11	Low
(van Loon-van Gaalen et al., 2021)	Systematic review	Checklist for Systematic Reviews and Research Syntheses	10/11	High
(Walton et al., 2023)	Scoping review	Checklist for Systematic Reviews and Research Syntheses	7/11	Moderate
(Bagkur et al., 2021)	Quasi-experimental	Checklist for Quasi-experimental Studies	3/9	Low
(Belanger & Arthur, 2024)	Mixed methods	Checklist for Quasi-experimental Studies (for the quantitative part of the study only)	5/9	Moderate
(Bergerot et al., 2023)	Prospective cohort	Checklist for Cohort Studies	2/11	Low
(Ceccato et al., 2021)	Cross-sectional	Checklist for Analytical Cross-sectional Studies	5/8	Moderate
(Corallo et al., 2023)	Quasi-experimental	Checklist for Quasi-experimental Studies	3/9	Low
(Cornejo Thumm et al., 2021)	Case study	Checklist for Case Reports	5/8	Moderate
(De Luca et al., 2021)	Randomized controlled trial	Critical Appraisal Tool for Assessment of Risk of Bias for Randomized Controlled Trials	6/13	Low
(Gani, Tan & King, 2023)	Prospective cohort	Checklist for Cohort Studies	5/11	Low

(Gayot et al., 2022)	Randomized controlled trial	Critical Appraisal Tool for Assessment of Risk of Bias for Randomized Controlled Trials	6/13	Low
(Ge et al., 2024)	Randomized controlled trial	Critical Appraisal Tool for Assessment of Risk of Bias for Randomized Controlled Trials	8/13	Moderate
(Gomez-Peralta et al., 2025)	Quasi-experimental	Checklist for Quasi-experimental Studies	4/9	Low
(Jeste et al., 2023)	Single-case experimental	Checklist for Quasi-experimental Studies	4/9	Low
(Lafaro et al., 2020)	Quasi-experimental	Checklist for Quasi-experimental Studies	3/9	Low
(Lai et al., 2020)	Quasi-experimental	Checklist for Quasi-experimental Studies	8/9	High
(Lang et al., 2021)	Quasi-experimental	Checklist for Quasi-experimental Studies	4/9	Low
(Laver et al., 2020)	Randomized controlled trial	Critical Appraisal Tool for Assessment of Risk of Bias for Randomized Controlled Trials	8/13	Moderate
(Lee, 2025)	Randomized controlled trial	Critical Appraisal Tool for Assessment of Risk of Bias for Randomized Controlled Trials	7/13	Moderate
(Leverentz et al., 2025)	Quasi-experimental	Checklist for Quasi-experimental Studies	5/9	Moderate
(Mancini et al., 2020)	Case study	Checklist for Case Reports	7/8	High

(Menengiç et al., 2021)	Randomized controlled trial	Critical Appraisal Tool for Assessment of Risk of Bias for Randomized Controlled Trials	7/13	Moderate
(Patanè et al., 2025)	Randomized controlled trial	Critical Appraisal Tool for Assessment of Risk of Bias for Randomized Controlled Trials	6/13	Low
(Pereira et al., 2020)	Case study	Checklist for Case Reports	7/8	High
(Persson, Lyth & Lind, 2020)	Quasi-experimental	Checklist for Quasi-experimental Studies	5/9	Moderate
(Rossetto et al., 2023)	Randomized controlled trial	Critical Appraisal Tool for Assessment of Risk of Bias for Randomized Controlled Trials	8/13	Moderate
(Sunner et al., 2023)	Randomized controlled trial	Critical Appraisal Tool for Assessment of Risk of Bias for Randomized Controlled Trials	6/13	Low
(Tsai et al., 2022)	Prospective cohort	Checklist for Cohort Studies	9/11	High
(Uemura et al., 2024)	Randomized controlled trial	Critical Appraisal Tool for Assessment of Risk of Bias for Randomized Controlled Trials	10/13	High
(Wu et al., 2023)	Quasi-experimental	Checklist for Quasi-experimental Studies	7/9	High
(Yerlikaya, Öniz & Özgùren, 2021)	Randomized controlled trial	Critical Appraisal Tool for Assessment of Risk of Bias for Randomized Controlled Trials	8/13	Moderate

(Yi & Yim, 2021)	Randomized controlled trial	Critical Appraisal Tool for Assessment of Risk of Bias for Randomized Controlled Trials	6/13	Low
(Yuan et al., 2024)	Randomized controlled trial	Critical Appraisal Tool for Assessment of Risk of Bias for Randomized Controlled Trials	8/13	Moderate

Supplementary Table 3. Main findings and characteristics of the selected reviews.

Authors (year)<br>Country<br>Number of studies	Objective	Intervention	Outcomes	Main findings
(Dawson et al., 2024)<br>Australia<br>11 studies	Evaluate impact of telehealth exercise on physical functioning in older adults with frailty, cognitive or mobility disability in any aged care settings excluding the hospital.	Exercise interventions delivered through synchronous or asynchronous telehealth vs. any comparator.	Mobility, balance, strength, falls, and QOL	Telehealth interventions showed small to moderate benefits on mobility, strength, and balance, but no significant effect on QOL. High acceptability/adherence linked to initial technological support, home-based convenience, and asynchronous program flexibility.
(Esfandiari, Miller & Ashe, 2021)<br>Canada<br>12 studies	Assess the effectiveness of telehealth on physical function and HRQOL in pre-frail or frail community-dwelling older adults.	Health programs delivered alone or in combination by telephone or video-calls, messages, email, apps, and DVD vs. no training or usual care.	Physical function, mobility, balance, PA, HRQOL, life satisfaction, falls, frailty status	Improvements in physical function, behavior, and mental QOL. Interventions were mostly telephone-based, focused on health promotion, and had low evidence quality.
(Eslami Jahromi & Ayatollahi, 2023)<br>Iran<br>10 studies	Investigate the impact of telecare interventions on QOL of older adults.	Any type of telecare interventions vs. traditional healthcare services.	QOL and its different dimensions (physical, mental, and general health, and social functioning)	Telemonitoring and telerehabilitation improved QOL in most studies. More user-centered designs are needed to ensure usability, acceptability, and cost-effectiveness.
(Markert et al., 2021)<br>USA<br>13 studies	Assess the effectiveness of health coaching remote interventions on delivering healthcare to older adults.	Health coaching programs combined with remote monitoring vs. any comparator.	Hospital admissions/re-admissions, mortality, hemoglobin level, weight, blood pressure, PA level, fatigue, QOL	Telehealth coaching, usually telephone-based, was effective in most studies. Further research is needed comparing human, automated, and hybrid coaching models for clinical and cost-effectiveness.
(van Loon-van Gaalen et al., 2021)<br>Netherlands<br>2 studies	Evaluate the effects of telephone follow-up after ED discharge on health-related outcomes in community-dwelling older adults	Telephone follow-up calls by a health care professional after ED discharge to an unassisted living environment vs. usual care or patient satisfaction survey.	Health service use (ED/hospital), physical function (daily living activities, independence), psychological outcomes (QOL, mood, satisfaction)	No benefits of scripted nurse-led telephone follow-up calls were found on health services utilization and discharge plan adherence.
(Walton et al., 2023)<br>USA<br>11 studies	Investigate the evidence of telehealth palliative care in nursing homes	Telehealth interventions between palliative care providers and NH residents and/or their surrogate decision makers vs. any comparator.	Symptom management, QOL, advance care planning, healthcare use, evaluation of care	Increased goals of care discussions and reduced acute care use. Video calls were the preferred telehealth modality.

Supplementary Table 4. Main findings and characteristics of the original articles

Authors (year)<br>Country	Study design	Aim of the study	Population characteristics	Intervention	Outcomes measured	Main findings
(Bagkur et al., 2021)<br>Cyprus	Quasi-experimental	Assess the impact of home-based interactive telerehabilitation on physical activity, sleep, and quality of life during confinement	Home-confined older adults during the COVID-19 pandemic<br>Mean age: 72.47 ± 5.58 years<br>(n = 23)	8 weeks<br>3x/week 40'<br>Exercise sessions via video-call + monitoring, led by a PT	BMI, physical activity (level/duration), sleep duration, TEE, PSQI, ESS, WHOQOL-OLD	Increase in physical activity levels and TEE, with improvements in sleep parameters and QOL
(Belanger & Arthur, 2024)<br>USA	Mixed methods	Examine the value of occupational therapy telehealth with a focus on health promotion and AiP concepts	Community-dwelling older adults<br>Mean age: 74.1 ± 6.8 years<br>(n = 10)	4 weeks<br>1x/week 60'<br>Online health promotion sessions delivered by OT	Pre/post-session survey scores	In average, the survey scores for each session increased by 37%
(Bergerot et al., 2023)<br>Brazil	Prospective cohort	Evaluate the impact of remote geriatric assessment before starting chemotherapy	Older adults diagnosed with any type of solid tumor scheduled to initiate chemotherapy<br>Mean age: 76 ± 7.2 years<br>(n = 56)	3 months<br>Psychologist-led video GA + referrals (geriatrician, psychologist, nutritionist)	IADL, GDS, FACT-G	Improvements in HR-QOL, physical functioning and depressive symptoms
(Ceccato et al., 2021)<br>Italy	Cross-sectional	Assess the efficacy of an emergency plan for continuing follow-up of endocrine outpatients during confinement	Outpatients with endocrine diseases<br>(n = 369)	8 weeks<br>1x/week 20'-30'<br>Initial phone triage followed by in-person or remote care (phone/email) with endocrinologist and nurse	Proportion of cancelled visits after triage.	Tele-endocrinology was applied to 63% of geriatric patients. Cancelled visits progressively decreased

(Corallo et al., 2023)<br>Italy	Quasi-experimental	Assess the impact of an inpatient telehealth intervention on cognitive and behavioral symptoms during COVID-19	Hospitalized patients with mild to moderate neurocognitive impairment<br>Mean age: 83.68 ± 10.16 years<br>(n = 28)	25 days<br>1x/day<br>Video calls with relatives	FIM, CDR, HAM-A, GDS, EQ-5D, NPI-Q, MMSE	Improvements in all variables. Higher QOL and mental health status
(Cornejo Thumm et al., 2021)<br>Israel	Case study	Evaluate the potential of a VR-based telerehabilitation intervention on a Parkinson's disease patient during confinement	Patient with Parkinson's disease<br>Age: 67 years	1 year<br>1x/week 15'-45'<br>VR-based treadmill training during video-call with increasing cognitive load + monitoring, led by a PT	Gait speed, walking endurance, ABC scale	Gait speed and endurance increased by 30% and 200%, respectively. Balance confidence improved from 55% to 70%
(De Luca et al., 2021)<br>Italy	RCT	Evaluate the effect of telemedicine on psychological, cognitive well-being and autonomy	Older adults living at home with ≥1 chronic medical or neuropsychiatric condition<br>EG (n = 30, 76.7 ± 8.3)<br>CG (n = 30, 78.2 ± 7.1)<br>(n = 60)	1 year<br>EG: 3x/week 45'<br>CG: 2-3x/month<br>Multi-specialist telemedicine care by video-calls and remote monitoring vs. in-person traditional care	GDS, BPRS, MMSE, BANSS, IADL, ADL, MNA	EG showed greater improvement in autonomy (IADL/ADL) and nutritional status vs. CG
(Gani, Tan & King, 2023)<br>Singapore	Prospective cohort	Evaluate the effect of telehealth on osteoporosis management following hip fracture.	Patients with a hip fracture<br>Mean age: 79.8 ± 8.23 years<br>(n = 537)	1 year<br>4x 15'<br>Nurse-led telephone calls with questionnaires on topics such as anti-osteoporosis medication and supplementation.	Percentage of patients on anti-osteoporosis treatment, calcium, and vitamin D supplementation	Increase in patients receiving anti-osteoporosis treatment (31.4% to 73.4%) and in calcium/vitamin D supplementation use (62.3% to 79.8%)
(Gayot et al., 2022)<br>France	RCT	Evaluate the impact of telemedicine in preventing unplanned hospitalization of residents in nursing homes in underserved areas	Nursing home residents with at least 2 chronic conditions<br>EG (n = 214, 87.2 ± 7.6)<br>CG (n = 212, 87.5 ± 7.42)<br>(n = 426)	1 year<br>3x 15'-30'<br>Preventative online teleconsultations with mini-GA, using connected devices if needed, led by a physician and supported by in-person geriatricians	Proportion of patients with unplanned hospitalizations, ADL, IADL, MNA, GDS, EQ-5D	Lower proportion of residents with unplanned hospitalizations in EG vs. CG
(Ge et al., 2024)<br>China	RCT	Compare the effectiveness of home physical therapy (HPTG) with telerehabilitation (TRG) in improving motor symptoms and quality of life	Patients with mild to moderate Parkinson's disease<br>TRG (n = 90, 69.12 ± 6.11)<br>HPTG (n = 100, 70.95 ± 5.74)<br>(n = 190)<br><br>*Age-stratified (<70/≥70)*	4 weeks<br>5x/week 40'-60'<br>Led by a PT, HPTG included 3 supervised home sessions + 2 independent sessions, and TRG was guided via app with videos and adverse event reporting	UPDRS-III, BBS, TUG, FTSST, FOGQ, gait, UPDRS-II, muscle strength, PDQ-39	HPTG showed greater effectiveness and adherence than TRG, particularly in older adults (≥70). Greater improvements in UPDRS-III, BBS, and TUG scores observed in HPTG.
(Gomez-Peralta et al., 2025)<br>Spain	Quasi-experimental	Evaluate the impact of a telehealth program incorporating continuous glucose monitoring (CGM) and a connected insulin pen cap (CIPC) on diabetes management in nursing homes	Nursing home residents with diabetes (mostly type-2)<br>Mean age: 87.7 ± 7.1 years<br>(n = 54)	10 weeks<br>CGM + insulin monitoring via CIPC, led by endocrinologist.<br>Nursing home staff received training and phone calls for treatment adjustments	Number of hypoglycemia events, glycemic control	Reduction in hypoglycemic events and insulin injections. Time below range <70 mg/dL decreased from 3.7% to 1.4%
(Jeste et al., 2023)<br>USA	Single-case experimental	Evaluate the effectiveness of a remotely delivered intervention focused on enhancing resilience and wisdom in reducing perceived stress and loneliness	Community-dwelling older adults without dementia<br>Mean age: 78.3 ± 7.8 years<br>(n = 20)	6 weeks<br>1x/week 60'<br>Video-call education sessions focused on cognitive, affective, and behavioral components, led by a therapist.	PSS-10, UCLA-3, CD-RISC-10, SD-WISE, APQ	Reduction in perceived stress and loneliness. Increase in resilience, happiness, wisdom, and positive ageing perceptions
(Lafaro et al., 2020)<br>Germany	Quasi-experimental	Assess the impact of a personalized telehealth intervention to enhance perioperative physical activity levels	Patients with lung or gastrointestinal cancer undergoing surgery<br>Median age: 73.0 years<br>(n = 34)	3-5 weeks<br>5x<br>Video-calls with PT/OT, from pre-operation to post-discharge, including walking/strength plans and coaching + pedometer step monitoring	6MWT, TUG, SPPB, daily steps	Improvements in mobility (6MWT, TUG) and lower extremity function (SPPB) from preoperative to 2–4 weeks post-discharge
(Lai et al., 2020)<br>China	Quasi-experimental	Compare the effectiveness of video and telephone-based (VTBG) and telephone only (TBG) telehealth in patients with dementia during confinement	Older adults with neurocognitive disorders<br>VTBG (n = 30, 72.9 ± 0.8)<br>TBG (n = 30, 72.7 ± 0.8)<br>(n = 60)	4 weeks<br>1-2x/week 30'<br>Both groups received phone calls on community living, healthy ageing, and well-being. VTBG received an additional weekly video call	MoCA, RMBPC, QOL-AD	MoCA and QOL-AD scores were significantly higher in VTBG at the end. MoCA declined in TBG but was maintained in VTBG
(Lang et al., 2021)<br>Germany	Quasi-experimental	Evaluate the impact of a telemonitoring intervention in HRQOL	Multimorbid older adults with and without depression and/or mild cognitive impairment<br>(n = 97)<br><br>*Age-stratified (65-74; 75-85, 86+)*	12 months<br>1-7x/week<br>Remote monitoring of vital signs and body weight with individualized frequency + phone calls and control questions if thresholds are exceeded	SF-12 (MCS/PCS), GDS	MCS improved over time and PCS remained unchanged
(Laver et al., 2020)<br>Australia	RCT	Compare the effectiveness of a dyadic intervention delivered via home visits (HVG) vs. telehealth (TG)	Community-dwelling older adults with dementia<br>HVG (n = 32, 80.47 ± 7.198)<br>TG (n = 31, 79.45 ± 6.52)<br>(n = 63)	16 weeks<br>8x 60'<br>OT-led sessions including assessment, education, stress management, and activity engagement.<br>HVG (8 home visits)<br>TG (2 home visits + 6 video calls)	CAFU, BOUP	There were no statistically significant differences between groups for CAFU or BOUP
(Lee, 2025)<br>South Korea	RCT	Compare the effectiveness of an exercise plan delivered remotely (REG) vs. in-person (IPEG), in improving physical function and reducing fall risk	Pre-frail older adults<br>REG (n = 30)<br>IPEG (n = 30)<br>CG (n=30)<br>(n = 90)	8 weeks<br>2x/week 50'<br>PT-led remote supervised via video: at home (REG) or in a senior center (IPEG). CG learned the plan and self-reported their physical activity	TUG, BBS, ABC scale, FTSST, 30CST, DGI, 10MWT, MFES	REG and IPEG showed greater improvements in balance, gait, strength, and fall efficacy vs. CG. No significant differences between REG and IPEG
(Leverentz et al., 2025)<br>USA	Quasi-experimental	Investigate the effects of a telehealth prevention program on the management of chronic conditions	Community-dwelling older adults with and at risk for chronic diseases<br>(n = 12)	6 weeks<br>1x/week 45'-60'<br>Video-call occupational therapy sessions focused on lifestyle and health promotion	GAS, SF-20	Great improvements in GAS scores

(Mancini et al., 2020) Italy	Case study	Assess the effects of a telenursing intervention on motor and non-motor symptoms in Parkinson's disease	Patient with Parkinson's disease<br>Age: 66 years	3 months<br>Telenursing disease management support via nurse-led telephone calls	NMSS	Marked reduction in falls. Improvement in non-motor symptoms
(Menengiç et al., 2021) Turkey	RCT	Investigate the effectiveness of telerehabilitation in Alzheimer's disease during confinement	Patients with early-middle stage of Alzheimer's disease<br>EG (n = 10, 77.7 ± 5.29)<br>CG (n = 10, 80.6 ± 6.11)<br>(n = 20)	6 weeks<br>4-5x/week 15'-40'<br>Motor and cognitive exercise treatment via video-call, led by a PT	MMSE, TUG, FTSST, OLST, ADL, FIM, GDS, BAS, WEMWBS	MMSE, TUG, FTSST increased significantly in EG vs. CG. Improvements also in anxiety, depression, and functional independence
(Patanè et al., 2025) Italy	RCT	Compare the effectiveness of a tele-prehabilitation plan delivered remotely with indirect electrostimulation (IEG) vs. home-based exercise (HBEG)	Patients eligible for lower limb arthroplasty<br>IEG (n = 20)<br>HBEG (n = 20)<br>(n = 40)	4 weeks<br>3x/week 30'<br>Video-call supervised sessions: IEG received PT-led indirect neuromuscular electrostimulation; HBEG followed an exercise plan, guided by a sports specialist	TUG, 30CST, 6MWT, OHS/OKS	Improvements in 6MWT scores in both groups. Greater QOL improvement in IEG vs. HBEG
(Pereira et al., 2020) Portugal	Case study	Assess the potential of a remote monitoring application for clinical follow-up and management of a hypertensive patient	Community-dwelling hypertensive patient<br>Age: 81 years	2 months<br>7x/week<br>Remote monitoring of blood pressure, heart rate, weight, and indoor environment via TV-based platform connected to medical devices	Blood pressure and heart rate control	Overall good blood pressure control, with occasional deviations motivating a close monitoring
(Persson, Lyth & Lind, 2020) Sweden	Quasi-experimental	Compare the impact of a telemonitoring intervention on preventing HRQOL deterioration in patients with COPD vs. CHF progression	Patients with COPD or CHF with ≥2 hospitalizations in the previous year<br>CHF (n = 58, 83 ± 7)<br>COPD (n = 36, 75 ± 6)<br>(n = 94)	1 year<br>7x/week<br>Self-reported health status via digital pen in a health diary + SMS alerts sent to healthcare staff for abnormal values or missing entries	EQ-5D, RAND-36, MLHFQ, SGRQ	Improvements in RAND-36. Disease specific HRQOL worsened among COPD group
(Rossetto et al., 2023) Italy	RCT	Evaluate the efficacy of a telerehabilitation intervention in patients within the Alzheimer's disease continuum during confinement	Patients with the Alzheimer's disease or mild cognitive impairment<br>EG (n = 15, 78.2 ± 3.95)<br>CG (n = 15, 77.13 ± 6.38)<br>(n = 30)	1 year<br>8x/week 15'-30'<br>Therapist led cognitive and motor activities: EG followed video tutorials + remote monitoring of activity results and vital parameters; CG followed written instructions	MoCA, verbal fluency test, TMT, FCSRT	Greater improvements in global cognitive function (particularly in language), executive function, and memory domains in the EG vs. CG
(Sunner et al., 2023) Australia	RCT	Evaluate the impact of incorporating teleconsultation into a telephone-based clinical support system for residential aged care facilities (RACF) on the rate of emergency department (ED) referrals during confinement	Nursing home residents<br>Man age: 84 ± 8 years<br>(n = 1,435)	14 months<br>ED nurse-led video-call providing clinical support to RACF staff during acute health events + follow-up phone calls	Rate of ED presentations during the service functioning period	29% reduction in the rate of ED presentations
(Tsai et al., 2022) Taiwan	Prospective cohort	Evaluate the effect of a cardiac telerehabilitation program on functional capacity, cardiac function, and readmission rates	Heart failure patients with reduced ejection fraction (LVEF <40%)<br>EG (n = 40, 75.6 ± 6.0)<br>CG (n = 41, 73.3 ± 5.0)<br>(n = 81)	6 months<br>7x/week 40'-60'<br>Multi-specialist telerehabilitation with remote monitoring of vital signs and exercise parameters via app + phone calls if abnormalities detected vs. traditional care	6MWT, LVEF, readmission rates	Greater improvements in functional capacity (6MWD) and LVEF. Decreased readmission rate
(Uemura et al., 2024) Japan	RCT	Investigate the impact of an active learning program on the prevention of physical activity decline, related behavioral skills, and self-efficacy	Community-dwelling older adults<br>EG (n = 15, 73.9)<br>CG (n = 14, 69.4)<br>(n = 29)	12 weeks<br>1x/week 90'<br>Active learning via video call + PA tracking (EG) vs. single cognitive health session + emailed content (CG)	PA amount and sedentary behaviors	Moderately higher maintenance effects on physical activity and sedentary behavior in the EG vs. CG
(Wu et al., 2023) China	Quasi-experimental	Compare the effectiveness of a telerehabilitation intervention (TRG) with telephone-based outpatient care (TG)	Patients who underwent total hip replacement<br>TRG (n = 43, 74.28 ± 5.06)<br>TG (n = 42, 72.00 ± 6.77)<br>(n = 85)	6 months<br>Multi-specialist program including video-calls + remote monitoring + messaging (TRG) vs. PT-led telephone-based follow-up (TG)	HHS, FIM, SAS, postoperative complications	TRG showed better outcomes vs. TG in HHS, FIM, and SAS scores. Complication rate was significantly lower in THG (14% vs 40.5%)
(Yerlikaya, Öniz & Özgùren, 2021) Cyprus	RCT	Compare the effectiveness of an interactive telerehabilitation program (ITHE) vs. non-supervised home exercise (NHE), on balance performance during confinement	Older adults with falling risk<br>ITHE (n = 18, 70.22 ± 5.53)<br>NHE (n = 16, 71.81 ± 6.57)<br>CG (n = 16, 75.62 ± 8.68)<br>(n = 50)	8 weeks<br>3x/week 40'<br>PT-led exercise program via video-call (ITHE) vs. non-supervised (NHE), vs. no exercise (CG)	BBS, TUG, TAI, WHOQOL-OLD	NHE and ITHE improved balance (BBS), mobility (TUG), and reduced fall risk. Increase of QOL in all groups, but no change in anxiety (TAI)
(Yi & Yim, 2021) South Korea	RCT	Assess the effectiveness of a remote exercise program on improving mental state, balance, and physical function during confinement	Community-dwelling older adults<br>EG (n = 35, 76.11 ± 6.31)<br>CG (n = 35, 77.31 ± 5.57)<br>(n = 70)	8 weeks<br>2x/week 40'<br>PT-led exercise program via video-call + motivation phone calls (EG) vs. no exercise (CG)	GDS, FTSST, grip strength, 10MWT, gait analysis, TUG, static balance test	EG showed higher improvements in all variables except dynamic balance (TUG) vs. CG
(Yuan et al., 2024) China	RCT	Investigate the effectiveness of a telenursing intervention in improving health status and QOL	Older adults with at least one chronic disease<br>EG (n = 39, 72.87 ± 5.02)<br>CG (n = 39, 71.51 ± 4.42)<br>(n = 78)	12 weeks<br>Nurse-led follow-up program including health promotion and guidance video-calls + remote monitoring (EG) vs. routine nursing (CG)	EQ-5D, SF-36, vital signs	Improvements in most components of EQ-5D and SF-36. Better management of blood pressure, glucose levels, weight, and sleep

Supplementary Table 5. Measurement variables found in the original articles.

Categories	Name of the assessment
Physical functioning and rehabilitation	Physical activity: duration, steps/day, TEE<br>Physical performance: TUG, FTSST, 30CST, 10MWT, 6MWT, SPPB, walking endurance<br>Muscle strength: handgrip, lower limb strength<br>Balance: ABC scale, BBS, OLST, static balance test<br>Gait: DGI, speed, step length, stride length<br>Falls: MFES
Clinical outcomes and disease management	Vital signs: blood pressure, heart rate, oxygen saturation, glycemia<br>Sleep: PSQI, ESS, sleep duration<br>Disease-specific outcomes: Parkinson's (FOGQ, UPDRS-II/-III, NMSS), Alzheimer's (BANSS), LVEF, HHS, post-operative complications<br>Plan adherence: cancelled endocrinology visits, anti-osteoporosis treatment, MNA, GAS<br>Healthcare utilization: proportion of unplanned hospitalizations, readmission rates
Cognitive and psychological	Cognitive functioning: MMSE, MoCA, CDR, NPI-Q, BPRS, RMBPC, BOUP, TMT, FCSRT, verbal fluency (phonemic/semantic)<br>Depression/anxiety: GDS, BAS, HAM-A, TAI<br>Stress/resilience: PSS-10, CD-RISC-10, SD-WISE<br>Social isolation/loneliness: UCLA-3<br>Ageing perceptions: APQ, SAS
QOL and well-being	HRQOL: EQ-5D, SF-12 (MCS, PCS), SF-20, FACT-G, QOL-AD, RAND-36, MLHFQ, OHS/OKS, PDQ-39, SGRQ<br>Overall QOL: WHOQOL-OLD<br>Well-being: WEMWBS<br>Independence: FIM, instrumental (IADL) or basic (ADL) activities of daily living, CAFU

10-MWT: 10-meter walk test; 30CST: 30-sec chair stand test; 6-MWT: 6-min walk test; ABC scale: Activities-Specific Balance Confidence Scale; APQ: Ageing Perceptions Questionnaire; BANSS: Bedford Alzheimer Nursing Severity Scale; BBS: Berg Balance Scale; BAS: Beck Anxiety Scale; BOUP: Behavioral Occurrence and Caregiver Upset; BPRS: Brief Psychiatric Rating Scale; CAFU: Caregiver Assessment of Functional Dependence; CD-RISC-10: Connor-Davidson Resilience Scale-10; CDR: Clinical Dementia Rating; DGI: Dynamic Gait Index; EQ-5D: EuroQol 5 Dimension; ESS: Epworth Sleepiness Scale; FACT-G: Functional Assessment of Cancer Therapy – General; FCSRT: Free and Cued Selective Reminding Test; FIM: Functional Independence Measure; FOGQ: Freezing of Gait Questionnaire; FTSST: five times sit-to-stand test; GAS: Goal Attainment Scale; GDS: Geriatric Depression Scale; HAM-A: Hamilton Anxiety Rating Scale; HHS: Harris Hip Score; LVEF: Left Ventricular Ejection Fraction; MFES: Modified Falls Efficacy Scale; MLHFQ: Minnesota Living with Heart Failure Questionnaire; MNA: Mini-Nutritional Assessment; MMSE: Mini-Mental State Examination; MoCA: Montreal Cognitive Assessment; NMSS: Non-Motor Symptoms Scale; NPI-Q: Neuropsychiatric Inventory Questionnaire; OHS/OKS: Oxford Hip/Knee Score; OLST: one-leg stand test; PDQ-39: Parkinson's Disease Questionnaire; PSQI: Pittsburgh Sleep Quality Index; PSS-10: Perceived Stress Scale; QOL-AD: Quality of Life in Alzheimer's Disease; RAND-36: 36-Item Health Survey; RMBPC: Revised Memory and Behavior Checklist; SAS: Self-rating Anxiety Scale; SD-WISE: San Diego Wisdom Scale; SGRQ: St. George's Respiratory Questionnaire; SF-12: 12-Item Short Form Health Survey (including scores for physical health (PCS) and mental health (MCS)); SF-20: 20-Item Short Form Health Survey; SPPB: Short Physical Performance Battery; TAI: Test Anxiety Inventory; TEE: total energy expenditure; TMT: Trail Making Test; TUG: Timed Up and Go; UCLA-3: Loneliness Scale; UPDRS-II/-III: Unified Parkinson's Disease Rating Scale Part II/Part III; WEMWBS: Warwick-Edinburgh Mental Wellbeing Scale; WHOQOL-OLD: WHO Quality of Life Instrument-Older Adults Module.
